# Computational Screening and Molecular Dynamic Simulation of Breast Cancer Associated Deleterious Non-Synonymous Single Nucleotide Polymorphisms in *TP53* Gene

**DOI:** 10.1371/journal.pone.0104242

**Published:** 2014-08-08

**Authors:** Kumaraswamy Naidu Chitrala, Suneetha Yeguvapalli

**Affiliations:** Department of Zoology, Sri Venkateswara University, Tirupati, Andhra Pradesh, India; The Institute of Cancer Research, United Kingdom

## Abstract

Breast cancer is one of the most common cancers among the women around the world. Several genes are known to be responsible for conferring the susceptibility to breast cancer. Among them, *TP53* is one of the major genetic risk factor which is known to be mutated in many of the breast tumor types. *TP53* mutations in breast cancer are known to be related to a poor prognosis and chemo resistance. This renders them as a promising molecular target for the treatment of breast cancer. In this study, we present a computational based screening and molecular dynamic simulation of breast cancer associated deleterious non-synonymous single nucleotide polymorphisms in *TP53*. We have predicted three deleterious coding non-synonymous single nucleotide polymorphisms rs11540654 (R110P), rs17849781 (P278A) and rs28934874 (P151T) in *TP53* with a phenotype in breast tumors using computational tools SIFT, Polyphen-2 and MutDB. We have performed molecular dynamics simulations to study the structural and dynamic effects of these *TP53* mutations in comparison to the wild-type protein. Results from our simulations revealed a detailed consequence of the mutations on the p53 DNA-binding core domain that may provide insight for therapeutic approaches in breast cancer.

## Introduction

One of the common malignancies and leading causes of cancer death faced by women around the world is breast cancer. Globally, the death rate of breast cancer has been rising around 2.5 lakhs in 1980 to 4.25 lakhs in 2010 [1]. Even in countries like China and India, its incidence is increased around 30% over the last decade whereas in Japan, Korea and Singapore it was doubled or even tripled [2]. According to National Cancer Institute (USA) statistics, estimated new cases of breast cancer in United States for the year 2013 is 232,340 in female and 2,240 in male whereas estimated breast cancer deaths are 39,620 in female and 410 in male. Some of the common risk factors for breast cancer can be broadly categorized into two types i.e., genetic and non genetic. Among these two risk factors, genetic risk factors constitute 5–10% of the breast cancer cases. Studies showed that fifty one variants in 40 genes are significantly associated with breast cancer risk and among them variants in six genes i.e., *BRCA1*, *BRCA2*, *TP53*, *PTEN*, *STK11* and *CDH1* show strong association whereas variants in four genes i.e., *ATM, CHEK2, BRIP1, PALB2* show moderate association and approximately 20 variants in other genes show weak association [3], [4].

Among the genes conferring high breast cancer risk, *TP53* is known to be mutated in 30% of the breast cancers cases with a higher frequency in some tumor subtypes [5]. *TP53* encodes p53, which is one of the most important tumor suppressor proteins in human cancers. p53 is a multi domain protein with 393 residues containing i) an acidic N-terminal transcription activation domain (1–44); ii) a proline-rich regulatory domain (62–94); iii) a central sequence-specific well conserved DNA-binding domain (110–292); iv) an oligomerization domain (325–363) and v) a C-terminal domain containing multiple regulatory signals (363–393) [6]. It functions as a tetramer. It is reported that 75% of all the mutations in *TP53* are missense, resulting in the substitution of a single amino acid with another and these mutations are predominantly distributed in the exons 4–9, encoding the DNA-binding domain of the protein [7].

Understanding how these single nucleotide polymorphisms (SNPs) affect the function of proteins is an important area of research and an efficient identification of such SNPs would be useful for SNP selection in genetic studies to understand the molecular basis of disease and predicting the effects of *in vitro* and *in vivo* mutagenesis experiments [8]. Among the SNPs, non-synonymous coding SNPs (nSNPs) are the one which are located in the coding regions resulting in an amino acid variation in the protein products of genes. They are believed to have a high impact on the phenotype [9]. In the present study, we have focused on the nSNPs in the coding region of *TP53* gene having an impact on breast cancer phenotype. We have explored the possible relationship between genetic mutation and phenotypic variation using different computational algorithm tools SIFT, PolyPhen-2 and Mutdb for prioritizing the deleterious breast cancer associated nSNPs from dbSNP datasets.

Molecular dynamics simulation (MDS) on the other hand is an important tool for understanding the effect of mutations on the protein structure, as it provides the information about the protein at atomic level on a reasonable time scale. Previously, several studies have utilized molecular dynamics to analyze the impact of mutations on *TP53* [10]–[14]. In order to check (i) whether the three mutants (R110P, P151T and P278A) have an impact on the conformation in the functionally significant regions of the p53C? (ii) Whether the mutant structures are deviating from the native p53C? (iii) Whether the mutants are changing the flexibility of the p53C? we have performed molecular dynamics simulations of WT and three mutants. Since, a significant fraction of p53 appears in apo state at physiological temperature and insufficient zinc is linked to misfolding, particularly in tumorigenic mutations, we performed both apo (Zinc-free state) and holo simulations and are presented here. Results showed that three mutants R110P, P151T and P278A are known to confer deleterious effect in the p53 DNA-binding core domain region (p53C). Overall, the objective of our study is to predict the breast cancer associated nSNPs and to further reveal the conformational flexibility of mutated apo and holo p53C through extensive molecular dynamic simulation.

## Materials and Methods

### Datasets

*TP53* SNPs were retrieved from dbSNP database (http://www.ncbi.nlm.nih.gov/projects/SNP/, Build 138; access date: May 13, 2013) [15] for our computational analysis.

### Prediction of deleterious coding *nSNPs*

We used both SIFT and Polyphen-2 to screen out the deleterious coding nSNPs from other SNPs for *TP53*. ‘Sorting Tolerant From Intolerant’ (SIFT) (http://sift.jcvi.org/; access date: May 15, 2013) is a multi-step algorithm that uses a sequence homology based approach [16] to predict whether an amino acid substitution in a protein will affect the protein function or not. For a given protein sequence, SIFT chooses the related proteins and obtains an alignment of them with the query and assigns scores to each residue. Scores ranging from 0–0.05 are considered to be intolerant or deleterious amino acid substitutions, where as scores ranging from 0.05–1 are considered be tolerant or neutral [17], [18]. We submitted our query in the form of dbSNP id to SIFT server. PolyPhen-2 (http://genetics.bwh.harvard.edu/pph2/; access date: May 18, 2013) [19] on the other hand is a web based server that predicts the functional significance of an allele replacement from its individual features by Naïve Bayes classifier. PolyPhen-2 prediction models were tested and trained using two pairs of datasets, one is HumanDiv compiled from all damaging alleles with known effects on the molecular function causing human Mendelian diseases, present in the UniProtKB database, together with differences between human proteins and their closely related mammalian homologs, assumed to be non-damaging and the other is HumVar, consisted of all human disease-causing mutations from UniProtKB, together with common human nsSNPs (MAF>1%) without annotated involvement in disease, which were treated as non-damaging. A mutation is appraised qualitatively, as benign, possibly or probably damaging based on pairs of false positive rate (FPR) thresholds, optimized separately for each model. Query was submitted in the form of dbSNP id to WHESS.db: a quick access to precomputed set of PolyPhen-2 predictions for whole human exome sequence space (WHESS).

### Prediction of phenotypic consequence for deleterious coding nSNPs

The phenotypic consequences of deleterious coding nSNPs were predicted using MutDB (http://www.mutdb.org/cgi-bin/mutdb.pl; access date: May 20, 2013), a tool that integrates publicly available databases of human genetic variation with molecular features and clinical phenotype data [20]. Gene symbol (‘*TP53*’) was used as a search term.

### Modeling nSNPs locations on protein structure and Molecular dynamics

**Figure 1:**
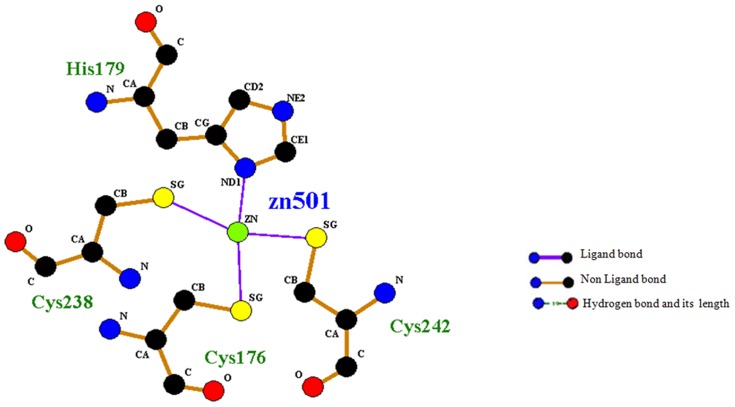
Ligplot showing the interactions of metal ion (Zn) with the amino acid residues of the protein. An atom of Zn bound with a tetra-co-ordinate geometry to three Cysteines (Cys 176, 238 and 242) and one Histidine (His 179).

To investigate the mechanism of structural consequences of the mutations on *TP53* we performed molecular dynamics. Initial coordinates were extracted from the crystal structure of p53 core domain in the absence of DNA (PDB ID: 2ocj, chain A; resolution 2.05 A°) [21]. All water molecules were removed from the crystal structure and the mutants (MTs) R110P, P151T, P278A were created by replacing the wild-type (WT) protein residue with its polymorphic residue using PyMOL [22]. Molecular dynamic analysis was performed at 37°C (physiological temperature) and neutral pH using GROMACS 4.5.3 (http://www.gromacs.org/) [23]–[25]. The p53 core domain contains Zn^2+^ that is essential for activity. A LIGPLOT [26] scheme of Zn^2+^ interaction in the crystal structure of p53 core domain (PDB ID: 2ocj, chain A; resolution 2.05 A°) was shown in the Fig. 1 given below. Zn^2+^ remains bound to p53 core domain at temperatures below 30°C and it rapidly dissociates at physiological temperature such that a significant fraction appears in the apo state [27]. Consequently, we focused on both apo and holo simulations of wild (WT) and mutant type (MT) p53 core domain and presented here. The system was solvated by adding explicit flexible SPC water [28] embedded in a cubic box and the walls were located ≥10 Å from all protein atoms. Cl^−^ counter ions (5, 3, 3, 5, 3, 5, 2 and 4 for holo WT, apo WT, apo P151T, holo P151T, apo P278A, holo P278A, apo R110P and holo R110P respectively) were added to neutralize the total charge of the system. The box size was set to 4.833 nm×4.027 nm×4.794 nm with box vectors 7.3 nm×7.3 nm×7.3 nm and box angles 90^0^ for each side. Each solvated structure was energy minimized for 50000 steps of steepest descent minimization terminating when maximum force is found smaller than 1000 KJ/mol^−1^/nm^−1^. After energy minimization, the system was subject to equilibration at constant temperature (300K) and pressure (1 bar) with a time step of 2 fs and non bonded pair list updated every five steps under the conditions of position restraints for heavy atoms and LINCS constraints [29] for all bonds. The temperature was kept constant using a Berendsen thermostat [30]. Electrostatic interactions were calculated using the particle mesh Ewald summation method [31]. Finally, eight (i.e., apo WT, holo WT, apo R110P, holo R110P, apo P151T, holo P151T, apo P278A and holo P278A respectively) 10 ns Molecular dynamics simulations (MDS) were performed.

### Analysis of Molecular dynamics trajectories

Comparative analysis of structural deviations in native and mutant structure such as root mean-square deviation (RMSD), root mean-square fluctuation (RMSF), solvent-accessible surface area (SASA), secondary structure calculation etc., were computed using g_rms, g_rmsf, g_sas and g_gyrate built in functions of GROMACS package. The average number of protein–solvent intermolecular hydrogen bonds was computed and analyzed using g_hbond. A cutoff radius of 0.35 nm was employed between the donor and the acceptor. Density map was plotted using g_densmap whereas average values of simulation output data was plotted using g_analyze of GROMACS respectively. Graphs were plotted using GRACE software (http://plasma-gate.weizmann.ac.il/Grace/).

### Principal component analysis

To analyze and visualize the overall motions in the simulations, essential dynamics (ED) was carried out according to the protocol in GROMACS software package [32]. Covariance analysis, also called principle component analysis or ED extracts the correlated motions of proteins to understand the motions that are most fundamental to its activity. ED is generally employed to characterize the large scale collective motions of a protein. Covariance matrices of both WT and MT simulations were constructed using the Cα atoms trajectory as it has been shown that they contain all the information for reasonable description of the protein large concerted motion [32]. We used gromacs utilities g_covar and g_anaeig for analyzing the trajectories.

## Results and Discussion

### SNP data set from dbSNP

**Figure 2:**
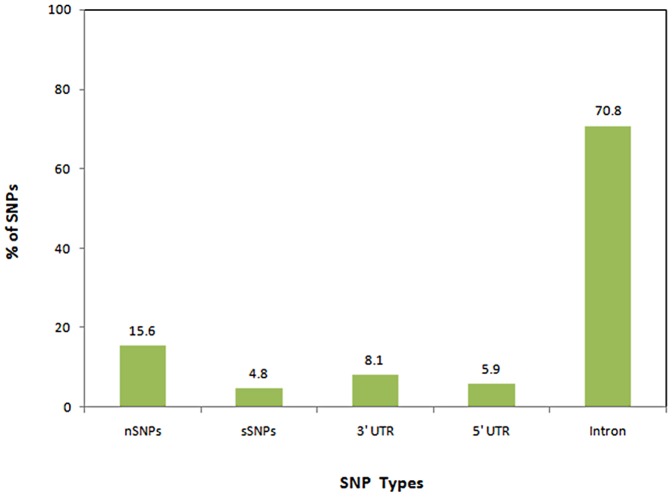
Distribution of *TP53* coding nonsynonymous SNPs (nSNPs), coding synonymous SNPs (sSNPs), 3′ UTR SNPs, 5′ UTR SNPs and intronic SNPs.

dbSNP database contain a total of 14613 SNPs for *TP53* gene, out of which 637 were found to be Human (active) SNPs (i.e., Active Human RS and not including those that have been merged). Among the 637 Human (active) SNPs, 100 were coding nSNPs, 31 were coding synonymous, 52 SNPs were in the mRNA 3′ UTR region, 38 were in the mRNA 5′ UTR region and 451 were in the intronic regions. It can be seen from the Fig. 2 that the vast majority of SNPs occur in the intronic region (70.8%) and more SNPs are nSNPs (15.6%) compared to synonymous SNPs (4.8%), SNPs occurring in the mRNA 3′ UTR (8.1%) and 5′ UTR (5.9%) regions. We selected coding nSNPs for our investigation.

### Deleterious nSNPs by SIFT server

Among the 100 nSNPs, from dbSNP 18 were found to be deleterious, with a tolerance index score of less than or equal to 0.05. We observed that among 18 deleterious nSNPs, 11 had a highly deleterious tolerance index score of 0.00 using orthologues and homologues in the protein alignment. Remaining 7 deleterious nSNPs had a tolerance index score of 0.01, 0.02, 0.03, 0.04 and 0.05 using orthologues and homologues in the protein alignment respectively (Table 1). Among 18 nSNPs that are predicted to be deleterious, three nSNPs showed a nucleotide change of A/T, five showed a change of A/G, one showed a change of C/G, five showed a change of C/T, one showed a change of G/T, one showed a change of C/G/T, one showed a change of A/G/T and one showed a change of A/C/T respectively. Compared to other nucleotide changes C/T and A/T change occurred the maximum number of times. Amino acid change on the other hand was majorly found to be from special amino acids to polar amino acids with uncharged R groups. Among them eight showed a change at the region of Arginine residues, three showed a change at the region of Proline residues, two showed a change at the region of Cysteine and the remaining five showed changes at the regions of Serine, Glycine, Leucine, Methionine, Glutamic Acid (Table 1).

**Table 1:** Prediction scores found to be functionally significant by SIFT server.

dbSNPID	Nucleotide Change	Amino acid change	Protein ID	Tolerance Index	
				Using orthologues in the Protein alignment	Using homologues in the Protein alignment
rs1042522	C/G	P72R	NP_000537	0.26	0.80
rs1642789	A/T	C339S	NP_001119585	0.05	0.00
rs1800371	C/T	P47S	NP_000537	0.49	0.06
rs2287499	C/G	R68G	NP_001137462	0.55	0.28
rs3021068	A/T	C341S	NP_001119586	0.00	0.00
**rs11540652**	**A/G**	**R248Q**	**NP_000537**	**0.00**	**0.04**
**rs11540654**	**C/G/T**	**R110P**	**NP_000537**	**0.04**	**0.00**
**rs17849781**	**C/G**	**P278A**	**NP_000537**	**0.00**	**0.05**
rs17880282	A/G	P11S	NP_001137462	0.00	0.00
rs17881470	G/T	S366A	NP_000537	0.77	0.01
rs17882252	A/G	E339K	NP_000537	0.15	0.00
**rs28934571**	**G/T**	**R249S**	**NP_000537**	**0.00**	**0.00**
**rs28934573**	**C/T**	**S241F**	**NP_000537**	**0.00**	**0.00**
**rs28934574**	**C/T**	**R282W**	**NP_000537**	**0.00**	**0.00**
**rs28934575**	**A/G/T**	**G245S**	**NP_000537**	**0.05**	**0.00**
**rs28934576**	**A/G**	**R273H**	**NP_000537**	**0.00**	**0.03**
**rs28934577**	**A/T**	**L257Q**	**NP_000537**	**0.00**	**0.00**
rs28934578	A/G	R175H	NP_000537	0.00	0.00
rs28934873	C/T	M133T	NP_000537	0.00	0.00
**rs28934874**	**A/C/T**	**P151T**	**NP_000537**	**0.02**	**0.00**
rs28934875	C/G	A138P	NP_000537	0.08	0.00
rs34067256	C/G	P136R	NP_001137462	0.31	0.93
rs35163653	A/G	V217M	NP_000537	0.06	0.00
rs35993958	C/G	G360A	NP_000537	0.76	0.27
rs55819519	C/T	R290H	NP_000537	0.29	0.09
**rs55832599**	**A/G**	**R267W**	**NP_000537**	**0.00**	**0.00**
rs56184981	C/T	N311S	NP_000537	0.74	0.39
rs72661117	A/G	D184N	NP_000537	0.25	0.00
rs72661119	A/G	N263D	NP_000537	0.30	0.01
rs80184930	A/G	S378P	NP_000537	0.10	0.00
rs111897235	A/G	A59T	NP_001137462	0.24	0.02
**rs112431538**	**C/T**	**E285K**	**NP_000537**	**0.01**	**0.00**
**rs121913343**	**C/T**	**R273C**	**NP_000537**	**0.00**	**0.00**

### Damaged nSNPs by PolyPhen-2 Server

To predict the functional significance of an allele replacement, 100 nSNPs analyzed by SIFT were submitted to PolyPhen-2 server. PolyPhen-2 qualitatively predicts whether a mutation is benign, possibly damaging, or probably damaging using two Bayesian probabilistic models, HumDiv and HumVar. For Mendelian disease diagnostics, the HumVar model is recommended as it should distinguish mutations with drastic effects from normal human variation whereas the HumDiv model is recommended for identifying variants where even mildly deleterious alleles are treated as damaging [19]. Among 100 nSNPs from dbSNP submitted to Polyphen-2, 37 were found to be possibly damaging, or probably damaging by both HumDiv and HumVar predictions whereas 2 were predicted as possibly damaging by only HumDiv, 36 were predicted as benign by both HumDiv and HumVar predictions and the remaining 25 were not scored as shown in the Table 2. Only SNPs that are predicted as possibly damaging or probably damaging by both HumDiv and HumVar predictions were considered for our study.

**Table 2:** Prediction scores found to be functionally significant by Polyphen-2 server.

dbSNPID	Nucleotide Change	Amino acid change	Protein ID	HDivPred	HDiv Prob	HVarPred	HVarProb
rs1042522	C/G	P72R	P04637	Benign	0.083	Benign	0.045
rs1642789	A/T	C339S	P04637	Benign	0	Benign	0
rs1800371	C/T	P47S	P04637	Benign	0.009	Benign	0.004
rs2287499	C/G	R68G	Q9BUR4	Benign	0	Benign	0
rs3021068	A/T	C341S	P04637	Benign	0.299	Benign	0.034
**rs11540652**	**A/G**	**R248Q**	**P04637**	**Probably damaging**	**1**	**Probably damaging**	**0.995**
**rs11540654**	**C/G/T**	**R110P**	**P04637**	**Probably damaging**	**0.911**	**Probably damaging**	**0.904**
**rs17849781**	**C/G**	**P278A**	**P04637**	**Probably damaging**	**0.999**	**Probably damaging**	**0.991**
rs17880282	A/G	P11S	Q9BUR4	Benign	0.011	Benign	0.01
rs17881470	G/T	S366A	P04637	Benign	0.001	Benign	0.002
rs17882252	A/G	E339K	P04637	Benign	0.188	Benign	0.127
**rs28934571**	**G/T**	**R249S**	**P04637**	**Probably damaging**	**0.997**	**Probably damaging**	**0.981**
**rs28934573**	**C/T**	**S241F**	**P04637**	**Probably damaging**	**1**	**Probably damaging**	**1**
**rs28934574**	**C/T**	**R282W**	**P04637**	**Probably damaging**	**1**	**Probably damaging**	**1**
**rs28934575**	**A/G/T**	**G245S**	**P04637**	**Probably damaging**	**1**	**Probably damaging**	**0.999**
**rs28934576**	**A/G**	**R273H**	**P04637**	**Possibly damaging**	**0.831**	**Possibly damaging**	**0.516**
**rs28934577**	**A/T**	**L257Q**	**P04637**	**Probably damaging**	**1**	**Probably damaging**	**0.999**
rs28934578	A/G	R175H	P04637	Possibly damaging	0.632	Benign	0.378
rs28934873	C/T	M133T	P04637	Benign	0.001	Benign	0.113
**rs28934874**	**A/C/T**	**P151T**	**P04637**	**Probably damaging**	**0.98**	**Possibly damaging**	**0.837**
rs28934875	C/G	A138P	P04637	Probably damaging	1	Probably damaging	0.998
rs34067256	C/G	P136R	Q9BUR4	Benign	0.007	Benign	0.005
rs35163653	A/G	V217M	P04637	Possibly damaging	0.687	Possibly damaging	0.578
rs35993958	C/G	G360A	P04637	Benign	0	Benign	0.001
rs55819519	C/T	R290H	P04637	Benign	0	Benign	0.002
**rs55832599**	**A/G**	**R267W**	**P04637**	**Probably damaging**	**0.979**	**Possibly damaging**	**0.868**
rs56184981	C/T	N311S	P04637	Benign	0	Benign	0
rs72661117	A/G	D184N	P04637	Benign	0.088	Benign	0.206
rs72661119	A/G	N263D	P04637	Benign	0.007	Benign	0.054
rs80184930	A/G	S378P	P04637	Benign	0.002	Benign	0.006
rs111897235	A/G	A59T	Q9BUR4	Benign	0.007	Benign	0.007
**rs112431538**	**C/T**	**E285K**	**P04637**	**Probably damaging**	**1**	**Probably damaging**	**0.994**
**rs121913343**	**C/T**	**R273C**	**P04637**	**Probably damaging**	**1**	**Probably damaging**	**0.999**
rs121912651	C/T	R248W	P04637	Probably damaging	1	Probably damaging	1
rs121912652	A/G	E258K	P04637	Probably damaging	1	Probably damaging	0.999
rs121912653	C/T	L252P	P04637	Probably damaging	1	Probably damaging	1
rs121912654	G/T	V157F	P04637	Probably damaging	0.999	Probably damaging	0.996
rs121912655	A/G	C242Y	P04637	Probably damaging	1	Probably damaging	1
rs121912656	A/G	G245D	P04637	Probably damaging	1	Probably damaging	0.999
rs121912657	G/T	V272L	P04637	Probably damaging	0.998	Probably damaging	0.966
rs121912659	G/T	G325V	P04637	Benign	0.045	Benign	0.189
rs121912660	G/T	R280T	P04637	Probably damaging	0.998	Probably damaging	0.984
rs121912661	C/G/T	L35F	P04637	Benign	0.068	Benign	0.06
rs121912662	C/T	L344P	P04637	Probably damaging	0.995	Probably damaging	0.967
rs121912663	A/T	K292I	P04637	Benign	0.017	Benign	0.164
rs121912664	A/G	R337H	P04637	Probably damaging	0.96	Possibly damaging	0.719
rs121912665	C/T	A189V	P04637	Possibly damaging	0.874	Possibly damaging	0.771
rs121912666	A/C	Y220S	P04637	Possibly damaging	0.995	Possibly damaging	0.977
rs121912667	A/T	E288V	P04637	Probably damaging	0.998	Probably damaging	0.998
rs137852789	A/G	G154S	P04637	Probably damaging	0.958	Possibly damaging	0.654
rs137852792	C/T	A129V	P04637	Benign	0	Benign	0
rs137853007	C/T	R145W	O96017	Probably damaging	1	Probably damaging	0.999
rs137854598	A/C	A102E	P42771	Probably damaging	1	Probably damaging	0.969
rs138729528	C/G	R175G	P04637	Probably damaging	0.999	Probably damaging	0.992
rs138983188	G/T	P223H	P04637	Probably damaging	1	Probably damaging	0.996
rs139002536	C/T	P45S	E9PMG4, Q9BUR4	Benign	0	Benign	0
rs140594263	C/T	A145V	Q9BUR4	Benign	0.002	Benign	0.004
rs140694361	C/T	P11L	E9PMG4, Q9BUR4	Benign	0.033	Benign	0.006
rs141402957	C/T	K351E	P04637	Possibly damaging	0.633	Possibly damaging	0.515
rs141850830	C/T	P86S	E9PMG4, Q9BUR4	Benign	0.079	Benign	0.021
rs144238223	A/G	E108G	E9PMG4	Possibly damaging	0.659	Benign	0.403
rs144340710	C/T	N235S	P04637	Benign	0.186	Benign	0.144
rs144386518	C/G	P58R	P04637	Benign	0.038	Benign	0.013
rs145151284	C/G	T312S	P04637	Benign	0	Benign	0.002
rs145760222	A/G	V63M	E9PMG4, Q9BUR4	Benign	0.015	Benign	0.007
rs146340390	A/G	P222L	P04637	Benign	0.042	Benign	0.068
rs147002414	A/G	P177S	P04637	Probably damaging	0.995	Probably damaging	0.964
rs147226406	A/G	A145T	Q9BUR4	Benign	0	Benign	0
rs148728256	A/C	E117A	E9PMG4	Benign	0.002	Benign	0.002
rs148924904	C/T	Y163C	P04637	Probably damaging	1	Probably damaging	1
rs149576018	C/G	R92G	E9PMG4, Q9BUR4	Benign	0	Benign	0.001
rs149633775	A/G	R283C	P04637	Benign	0.089	Benign	0.087
rs150282629	G/T	S16A	E9PMG4, Q9BUR4	Benign	0.001	Benign	0.002
rs150607408	C/T	S185N	P04637	Benign	0	Benign	0.002
rs150842067	A/G	L383F	P04637	Possibly damaging	0.865	Possibly damaging	0.594

### Breast Cancer related mutations by Mutdb database

Results from both SIFT and Polyphen-2 analysis showed that among 100 nSNPs, only 15 SNPs were predicted to be deleterious or damaging on protein function. These 15 SNPs were submitted to Mutdb to confirm that they confer a breast cancer phenotype or not. Results showed that 3 SNP mutations i.e., rs11540654 (R110P), rs17849781 (P278A) and rs28934874 (P151T) are known to have a phenotype in Breast tumors (Table 3).

**Table 3:** Phenotype information of *TP53* variants.

dbSNPID	Nucleotide change	Amino acid change	Prediction		
			SIFT	Polyphen-2	Phenotype (Mutdb)
rs11540652	A/G	R248Q	Intolerant	Probably damaging	In LFS and many types of tumors
**rs11540654**	**C/G/T**	**R110P**	**Intolerant**	**Probably damaging**	**In a breast tumor**
**rs17849781**	**C/G**	**P278A**	**Potentially intolerant**	**Probably damaging**	**In a breast tumor**
rs28934571	G/T	R249S	Intolerant	Probably damaging	In many types of tumors
rs28934573	C/T	S241F	Intolerant	Probably damaging	In a colon tumor
rs28934574	C/T	R282W	Intolerant	Probably damaging	In esophageal adeno carcinoma and many types of tumors
rs28934575	A/G/T	G245S	Potentially intolerant	Probably damaging	In esophageal adeno carcinoma and many types of tumors
rs28934576	A/G	R273H	Intolerant	Possibly damaging	In LFS, colon and esophagus tumors
rs28934577	A/T	L257Q	Intolerant	Probably damaging	Nil
**rs28934874**	**A/C/T**	**P151T**	**Intolerant**	**Possibly damaging**	**In a breast tumor**
rs28934875	C/G	A138P	Potentially intolerant	Probably damaging	In a lung tumor
rs35163653	A/G	V217M	Potentially intolerant	Possibly damaging	Nil
rs55832599	A/G	R267W	Intolerant	Probably damaging	Nil
rs112431538	C/T	E285K	Intolerant	Probably damaging	Nil
rs121913343	C/T	R273C	Intolerant	Probably damaging	Nil

### Molecular dynamics simulation studies

**Figure 3:**
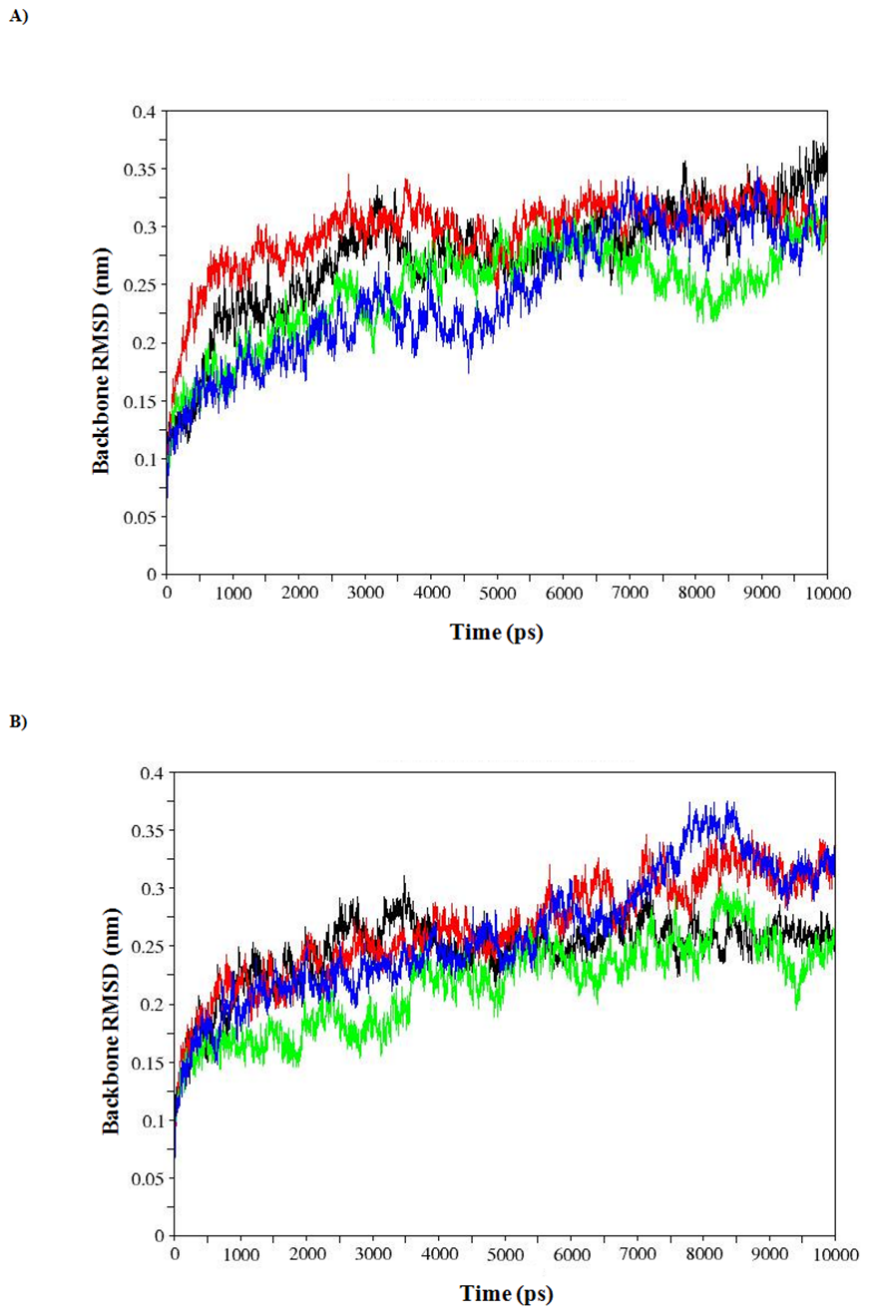
Backbone rmsd values for WT, R110P, P151T and P278A during A) Apo simulations B) Holo simulations for p53C. Black: WT, red: R110P, green: P151T and blue: P278A.

Results from the calculations of RMSD for backbone and Cα atoms, root mean square fluctuation (RMSF) for Cα atoms, radius of gyration (Rg) for Cα atoms and protein for apo and holo WT, R110P, P151T, P278A MDS were presented in the Table 4. To analyze the impact of MTs on the p53C, we have examined the RMSD values. The calculated RMSDs of the backbone atoms in apo and holo WT, R110P, P151T, P278A with respect to the starting structure during the 10-ns MDS as a function of time were plotted in the Fig. 3a, b. During the apo simulations, backbone RMSDs of the WT and MT structures showed a sharp increase in the first 3 ns followed by equilibrium around 6 ns and a sudden decrease around 7.5 ns for P151T, 9.5 ns for P278A and R110P (Fig. 3a) whereas in the case of holo simulations a different pattern was observed. A sharp increase is shown during the first 3.5 ns followed by equilibrium around 4 ns and a sudden increase around 7.5 ns for P151T, P278A and a sudden decrease around 9.5 ns for R110P. A comparison of average backbone RMSD values showed the following order of structural deviations (Table 4): apo; R110P > WT > P151T  =  P278A, holo; R110P  =  P278A > WT >P151T. A variation in the average backbone RMSD values of WT and MTs lead to the conclusion that these mutations could affect the dynamic behavior of p53C, thus provides a suitable basis for further analyses.

**Table 4:** Time averaged structural properties calculated for WT, R110P, P151T, P278A holo [with Zn^2+^ ion present] and apo [with Zn^2+^ ion absent] p53 core domain.

	initial WT		R110P		P151T		P278A	
	apo	holo	apo	holo	apo	holo	apo	holo
Backbone rmsd (nm)	0.27 (0.04)	0.24 (0.02)	0.29 (0.03)	0.26 (0.04)	0.24 (0.04)	0.21 (0.03)	0.24 (0.05)	0.26 (0.05)
Cα-rmsd (nm)	0.28 (0.04)	0.25 (0.03)	0.30 (0.03)	0.27 (0.04)	0.25 (0.04)	0.22 (0.03)	0.24 (0.05)	0.26 (0.05)
Cα-rmsf (nm)	0.15 (0.08)	0.12 (0.07)	0.13 (0.08)	0.13 (0.07)	0.13 (0.07)	0.12 (0.06)	0.14 (0.08)	0.14 (0.09)
Rg- Cα (nm)	1.64 (0.01)	1.64 (0.01)	1.62 (0.09)	1.63 (0.08)	1.64 (0.08)	1.63 (0.07)	1.63 (0.01)	1.64 (0.01)
Rg-protein (nm)	1.67 (0.01)	1.67 (0.01)	1.65 (0.01)	1.66 (0.08)	1.66 (0.08)	1.66 (0.08)	1.66 (0.01)	1.66 (0.0)
Trace of the diagonalized covariance matrix (nm^2^)	5.67543	4.51117	4.86401	4.81546	4.55228	4.11307	5.70146	5.5473

Mean values—averaged over the trajectory or over the number of residues employed at each calculation—with standard deviations given in parentheses. Cα-rmsd: Cα-root-mean-square deviation, Rg: Radius of gyration; SASA: Solvent Accessible Surface Area

**Figure 4:**
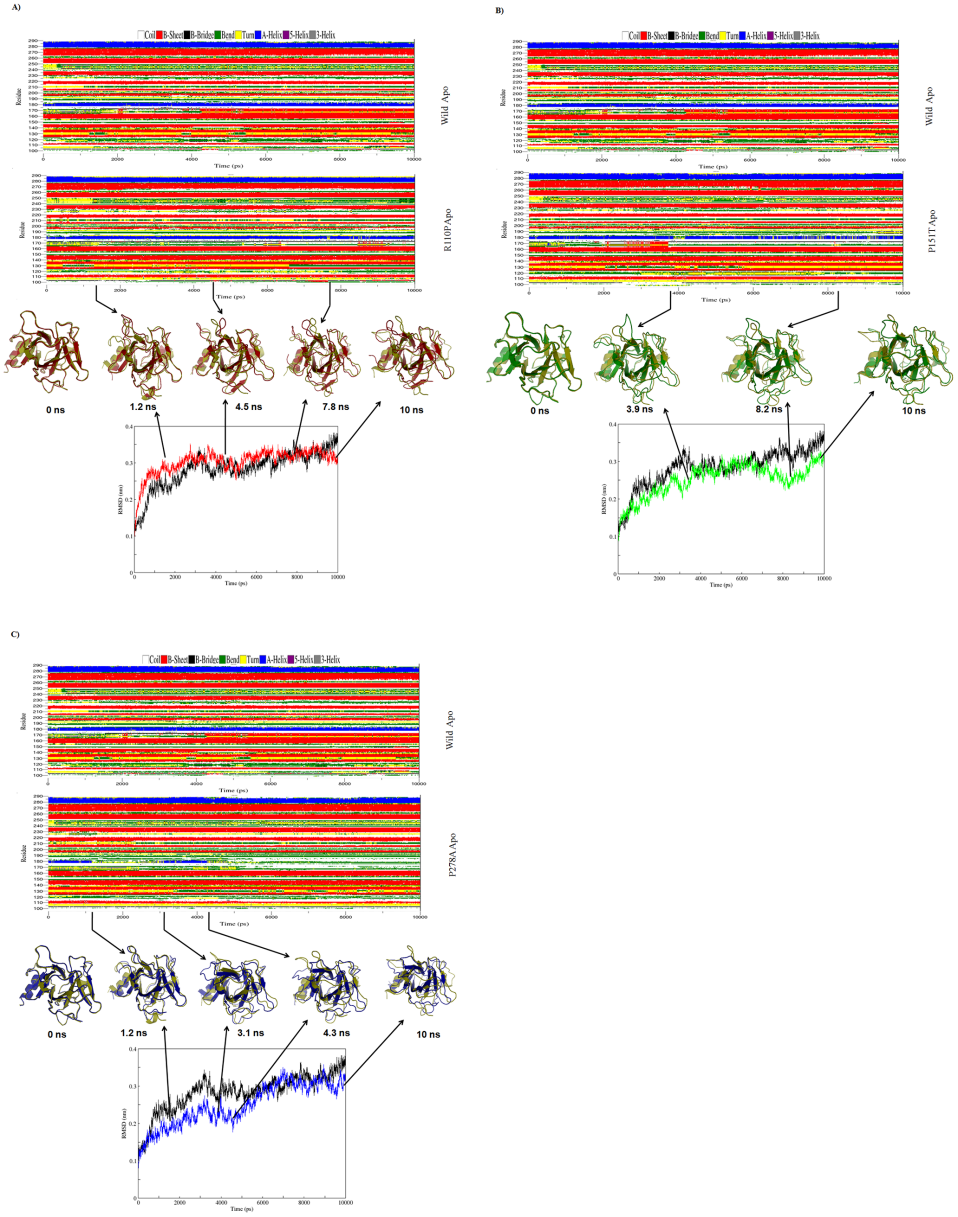
RMSD and DSSP changes in WT and MT structures during the 10-ns apo MDS. A) Figure shown at the top represents WT and R110P DSSP plot. In the middle superimposed WT and R110P structures are shown. Yellow: WT, red: R110P. At the bottom, the Cα RMSD plot is shown as a function of time. Black: WT, red: R110P. B) Figure shown at the top represents WT and P151T DSSP plot. In the middle superimposed WT and P151T structures are shown. Yellow: WT, green: P151T. At the bottom, the Cα RMSD plot is shown as a function of time. Black: WT, green: P151T. C) Figure shown at the top represents WT and P278A DSSP plot. In the middle superimposed WT and P278A structures are shown. Yellow: WT, blue: P278A. At the bottom, the Cα RMSD plot is shown as a function of time. Black: WT, blue: P278A.

**Figure 5:**
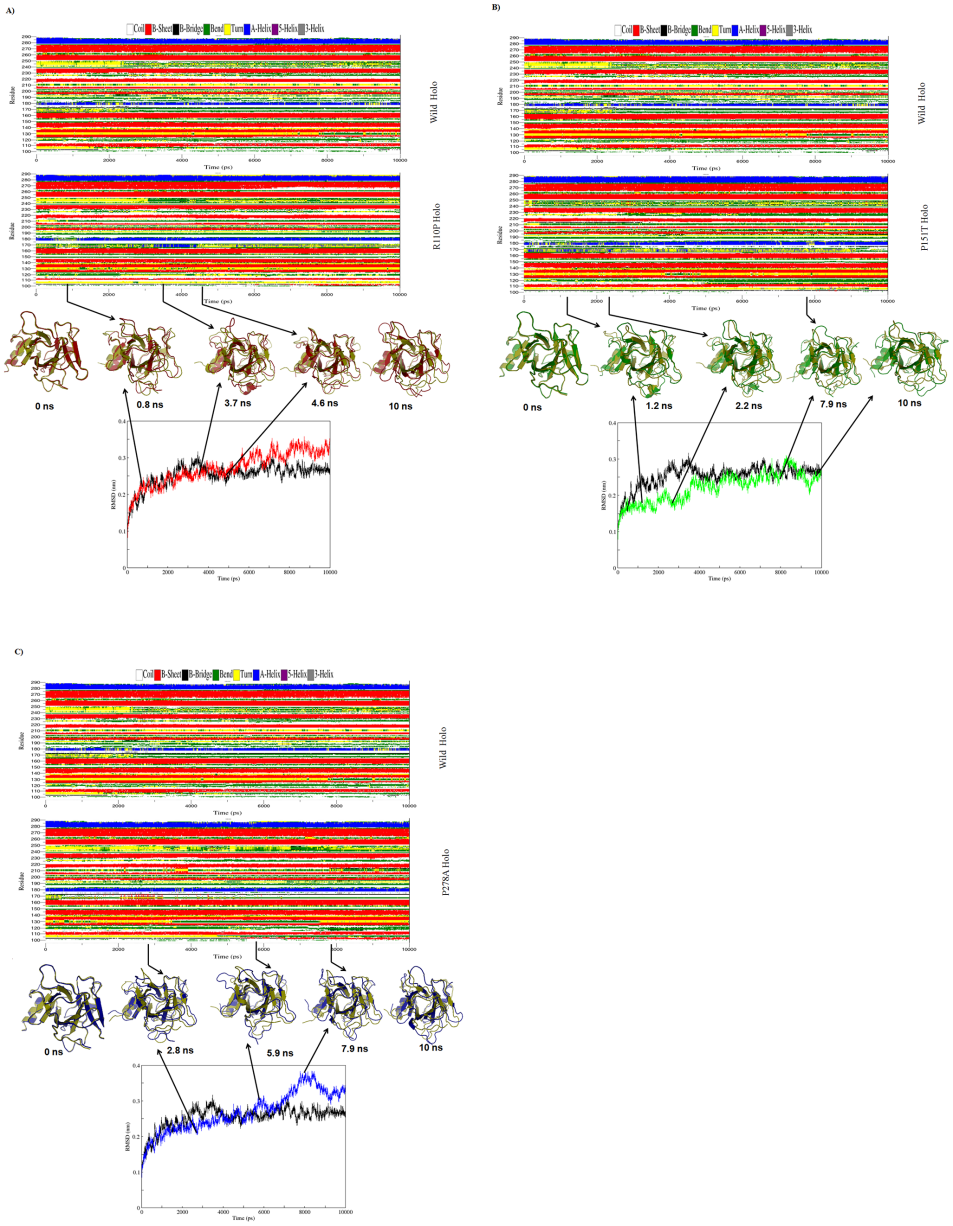
RMSD and DSSP changes in WT and MT structures during the 10 –ns holo MDS. A) Figure shown at the top represents WT and R110P DSSP plot. In the middle superimposed WT and R110P structures are shown. Yellow: WT, red: R110P. At the bottom, the Cα RMSD plot is shown as a function of time. Black: WT, red: R110P. B) Figure shown at the top represents WT and P151T DSSP plot. In the middle superimposed WT and P151T structures are shown. Yellow: WT, green: P151T. At the bottom, the Cα RMSD plot is shown as a function of time. Black: WT, green: P151T. C) Figure shown at the top represents WT and P278A DSSP plot. In the middle superimposed WT and P278A structures are shown. Yellow: WT, blue: P278A. At the bottom, the Cα RMSD plot is shown as a function of time. Black: WT, blue: P278A.

Since, RMSD of the Cα atoms is a central origin to compute the protein system [33], we have calculated the respective Cα RMSDs for both apo and holo simulations and plotted in the Fig. 4, 5. During the apo simulations, Cα-RMSD of R110P showed a sharp increase in the initial 2.5 ns followed by equilibrium around 4 ns and a sudden decrease after 9 ns (Fig. 4a). However, P151T and P278A showed a different trend of Cα-RMSD, with an equilibrium around 4 ns and a sudden decrease around 7 ns for P151T (Fig. 4b) whereas an equilibrium around 6 ns and a sudden decrease around 9 ns for P278A (Fig. 4c). During holo simulations on the other hand, Cα-RMSD of R110P showed a less variation in the initial 2 ns followed by equilibrium around 4 ns and a sudden decrease after 5 ns (Fig. 5a). However, P151T and P278A showed a different trend of Cα-RMSD, with an equilibrium around 5 ns and a sudden decrease around 9.5 ns for P151T (Fig. 5b) whereas an equilibrium around 4 ns and a sudden increase around 7 ns for P278A (Fig. 5c). A comparison of average Cα-RMSD values showed the following order of structural deviations (Table 4): apo; R110P > WT > P151T > P278A, holo; R110P > P278A > WT > P151T. These results indicate that a greatest change was observed in the R110P compared to the other mutants in both apo and holo simulations.

In order to analyze the change in secondary structure patterns in WT and MTs, we applied the software tool DSSP (Database of Secondary Structure in Proteins) by Kabsch and Sander [34], which employs H-bonding patterns and various other geometrical features to assign secondary structure labels to the residues of a protein. We have plotted the secondary structure patterns between WT and MTs of both apo and holo simulations and also superimposed their respective structures at the beginning of the simulation and for specific time steps where the conformational drifts occurred at a higher range (Fig. 4,5). Analysis of time dependent secondary structure fluctuations through DSSP analysis showed a conformational drift from β-sheets to bend form between the residues 165–175 in R110P and α-helix to bend form for the 180^th^ residue in P151T and P278A during the apo simulations and a conformational drift from α-helix to bend for the 180^th^ residue in R110P, P151T and turn to bend form for the 130^th^ residue in P278A during the holo simulations (Fig. 4, 5). The conformational changes support our previous results obtained from RMSD analysis that major change occurred in the R110P (Fig. 2,4,5).

**Figure 6:**
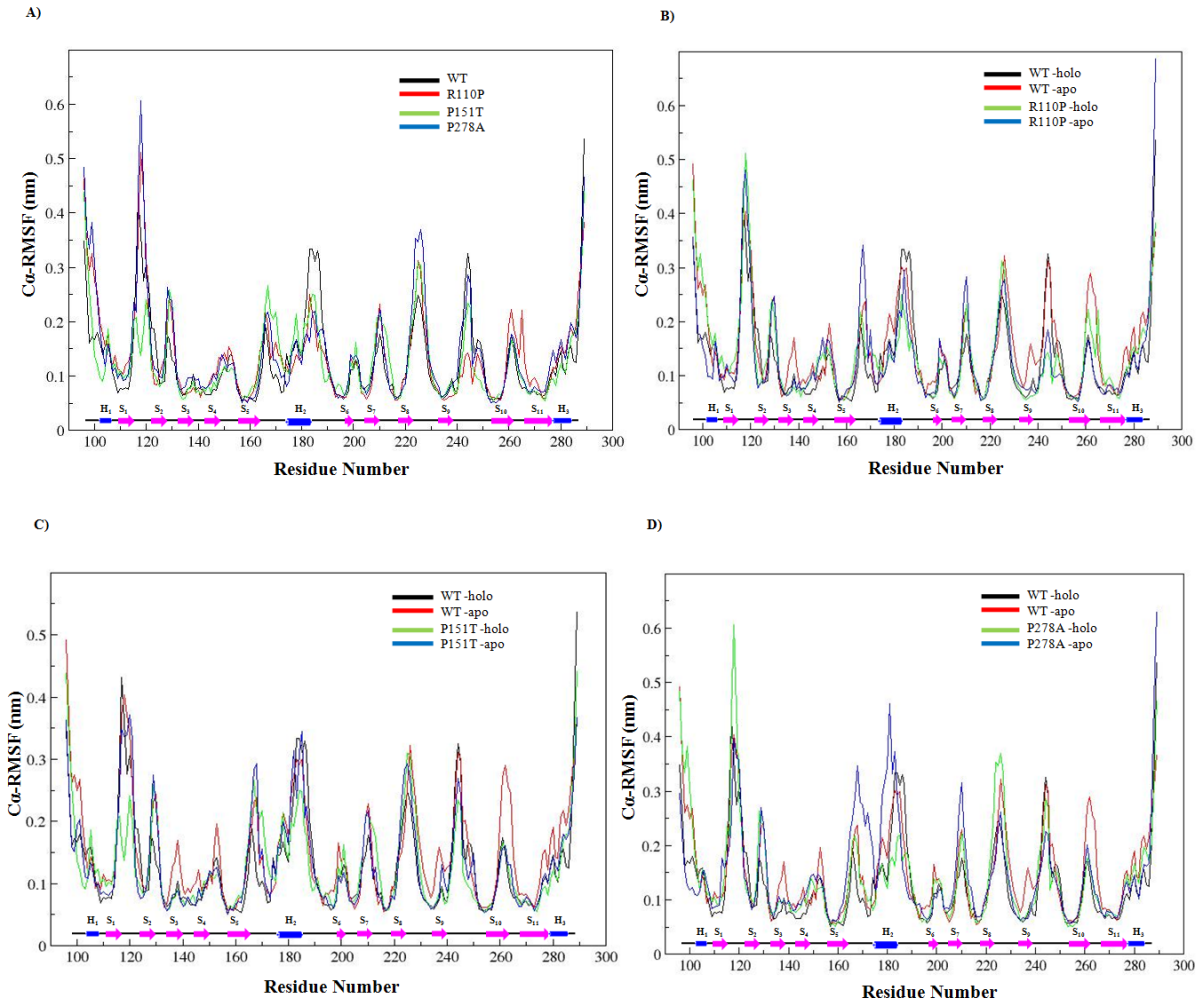
RMSF of Cα atoms as a function of amino acids.

In order to understand how the mutants affect the dynamic behaviour of the residues and to examine the cause of conformational drifts observed in RMSD and secondary structure patterns, Cα-root mean square fluctuation (Cα-RMSF) of WT and MT amino acid residues were calculated and plotted in the Fig. 6a-d. Except P151T, in all cases the MT holo simulations had higher average Cα-RMSFs than the WT holo simulations (Fig. 6a) (Table 4). In the apo and holo WT, more than 50% of the residues have RMSF values >0.1 nm (Table 5) indicating a higher level of fluctuation. During the holo simulations, all the MTs showed a larger percentage of residues (i.e., Cα residues and residues in the protein core comprising secondary structural elements) with RMSF values >0.1 nm whereas during apo simulations less percentage of residues showed RMSF values >0.1 nm compared to the WT. These results indicate that compared to apo, holo simulations are associated with increase in flexibilities in MTs. Among the holo MTs, R110P have a higher percentage of residues with RMSF >0.1 nm thus indicating a higher effect on the overall flexibility of the p53C.

**Table 5:** Percentage wise distribution of Cα RMSF values.

	RMSF (nm)			
	All Cα		Sec-str Cα	
	≤0.1 nm	>0.1 nm	≤0.1 nm	>1 nm
WT apo	34	66	26	20
WT holo	47	52	34	13
R110P apo	47	52	31	9
R110P holo	38	61	27	18
P151T apo	46	53	32	12
P151T holo	44	55	29	15
P278A apo	35	64	27	17
P278A holo	41	58	29	15

Further, comparison of the regional flexibilities of the MTs showed a characteristic increase and decrease of the flexibility in certain loops, helices and β-sheets (Table 6). Strand S10 showed a consistently low fluctuation across all the simulations whereas higher fluctuations around the Zn^2+^ binding residues, C176, H179, C238 and C242 were observed in apo WT and MTs compared to the holo WT simulations (Table 7). Loop regions on the other hand showed a larger fluctuation in both WT and MTs. Changes in the loops L3, L11, L12 and H2 helix contributed to a higher value of Cα-RMSF in the MTs with a larger portion of the L3 loop, S10 strand and H2 helix shifted far from its starting position (Fig. 6a). Compared to the WT simulations, the fluctuations observed were noticeably increased around the loops L3 and L7 in the R110P (Fig. 6b) whereas in P151T noticeable increase in fluctuations were observed around the loops L4 and L7 (Fig. 6c). P278A on the other hand showed an increase in fluctuations at the loops L3, L7, L11 and H2 helix regions (Fig. 6d). Results from the analysis of regional flexibilities indicate that all the three MTs R110P, P151T and P278A will affect the overall flexibility of p53C.

**Table 6:** Regions (α-helices, β-sheets, and loops) showing an average increase or decrease of RMSF in the MTs compared to the WT; RMSF of a particular structure is taken to be increased or decreased if there is an average change in RMSF of >0.03 nm in at least ≥50% of its residues.

	Increase	Decrease
R110P apo	L_4_, S_4_, L_6_, L_7_, L_10_,	L_3_, L_8_, L_12_,
R110P holo	L_1_, S_1_, L_3_, S_2_, L_4_, L_7_, L_10_, L_13_,	H_2_, L_8_,
P151T apo	L_4_, L_7_, H_2_,	L_3_, L_8_,
P151T holo	L_1_, L_2_, L_4_, L_7_, L_10_, L_11_,	L_3_, L_8_, L_12_,
P278A apo	L_3_, L_4_, L_7_, H_2_, L_10_,	L_1_, L_8_,
P278A holo	L_1_, L_4_, L_7_, L_10_, L_11_,	L_3_, L_8_,

**Table 7:** Cα RMSF values (nm) at Zn^2+^ binding residues, C176, H179, C238 and C242.

Residue	WT holo	WT apo	R110 apo	R110 holo	P151T apo	P151T holo	P278A apo	P278A holo
C176	0.1452	0.1598	0.1021	0.1056	0.1588	0.1458	0.1247	0.1331
H179	0.148	0.1985	0.1322	0.1301	0.1846	0.1523	0.3181	0.1354
C238	0.0959	0.1383	0.0726	0.0619	0.1311	0.0893	0.0809	0.0814
C242	0.1733	0.1553	0.1042	0.0978	0.1578	0.1367	0.1539	0.1983

**Figure 7:**
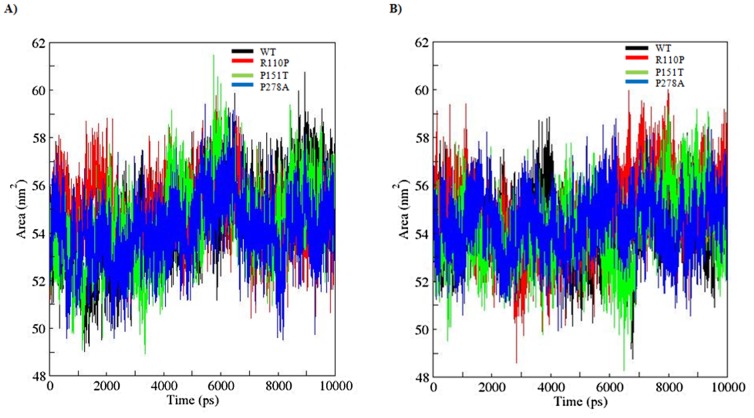
Solvent-accessible surface area (SASA) of WT and MT versus time during A) Apo B) Holo simulations for p53C. Black: WT, red: R110P, green: P151T and blue: P278A.

**Figure 8:**
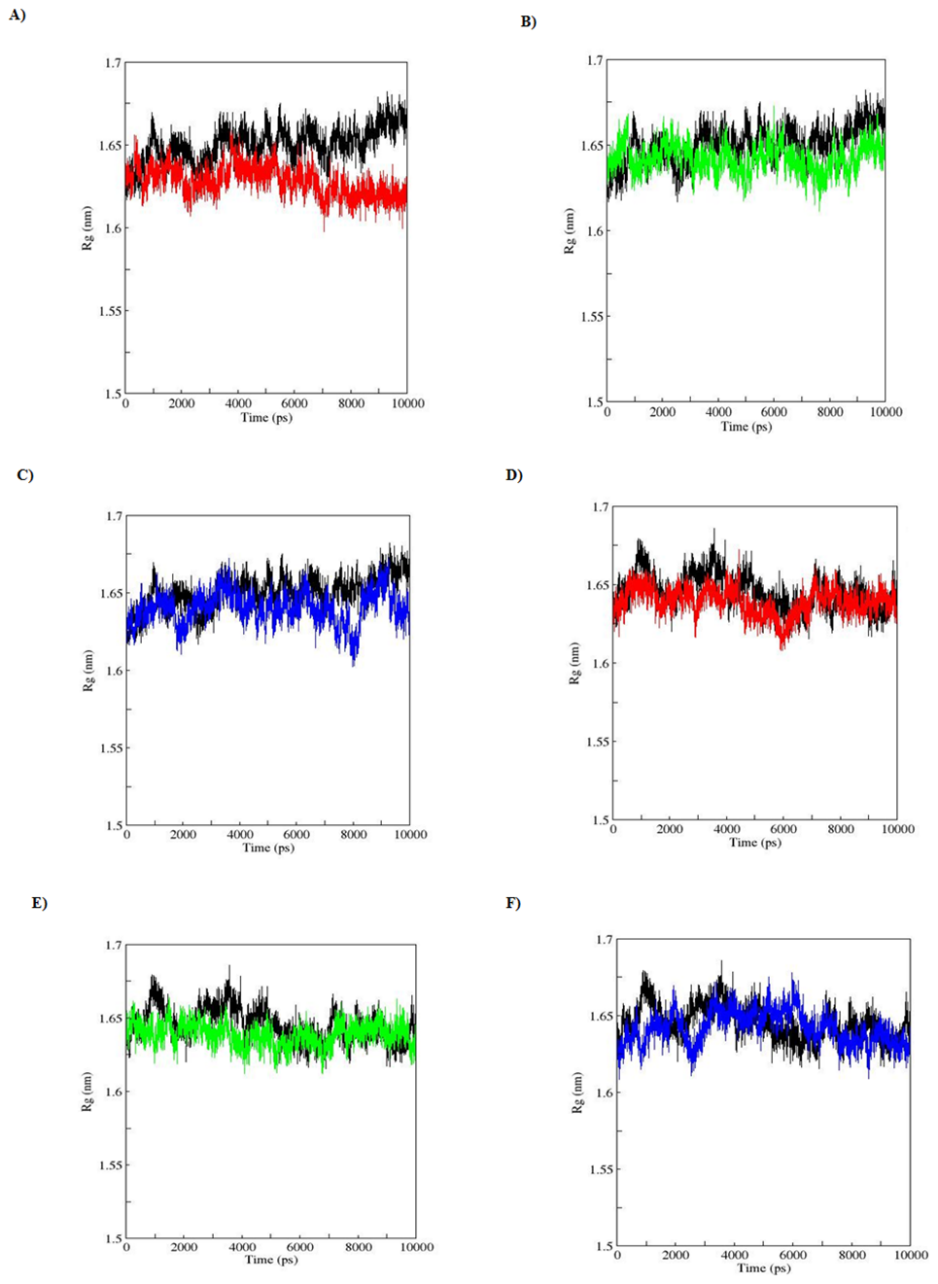
Radius of gyration of Cα atoms during a 10-ns MDS for WT and MT p53C versus time. A), B), C) represent apo simulation D), E), F) represent holo simulation. Black: WT, red: R110P, green: P151T and blue: P278A.

**Figure 9:**
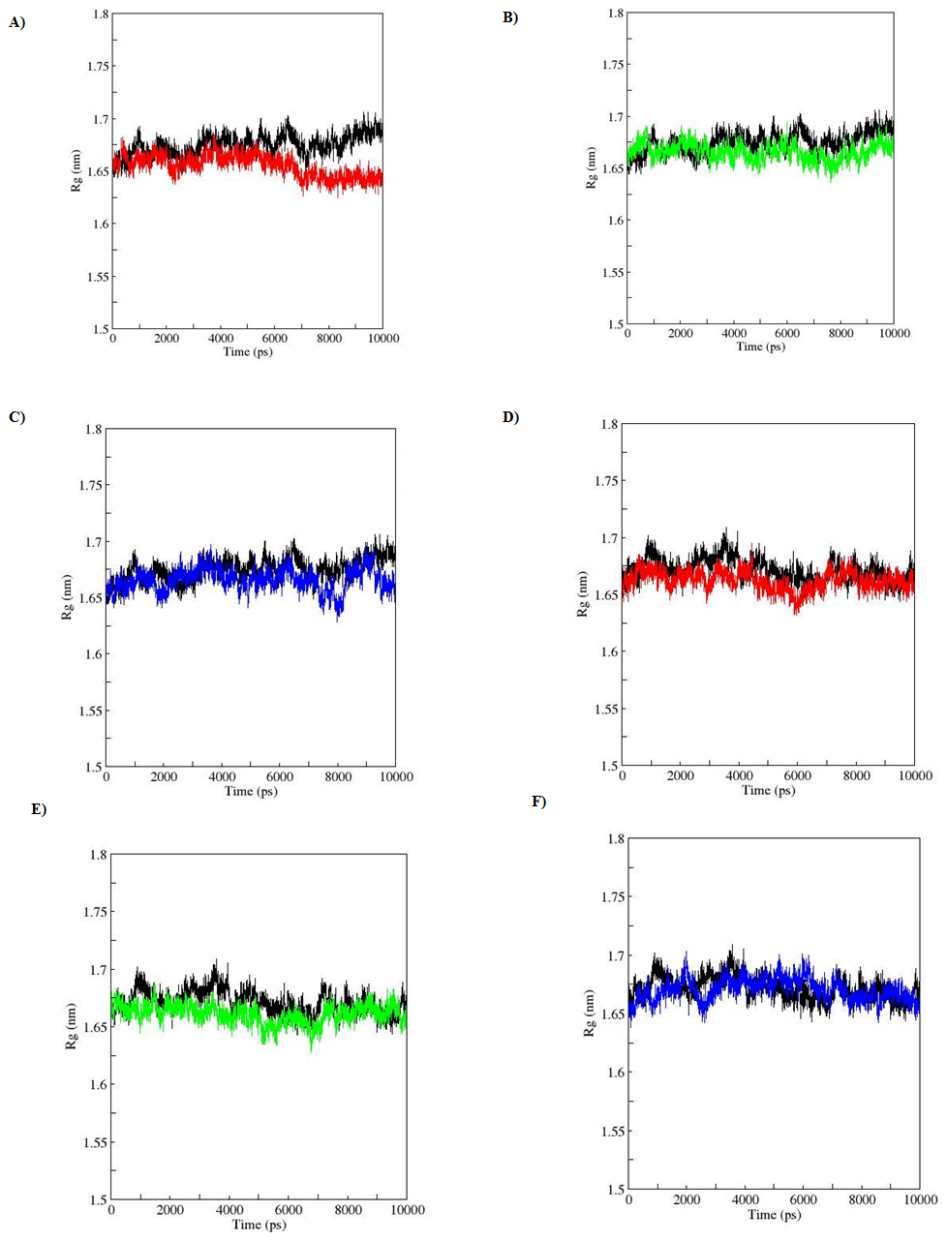
Radius of gyration of Protein during a 10-ns MDS for WT and MT p53C versus time. A), B), C) represent apo simulation D), E), F) represent holo simulation. Black: WT, red: R110P, green: P151T and blue: P278A.

SASA is a geometric measure of the extent to which an amino acid interacts with its environment (the solvent and the protein core). It is naturally proportional to the degree to which an amino acid is exposed to these environments [35]. A rise or fall in the SASA designates the change in exposed amino acid residues thereby affecting the tertiary structure of a protein. Results from the analysis of SASA for apo and holo simulations showed a variation among the WT and MTs (Fig. 7a-b). MTs (apo; R110P:117.5916, P151T:118.8266, P278:118.7204, holo; R110P:118.1768, P151T:118.3956, P278A:118.7466) showed a lesser average total SASA compared to the WT (apo; 119.7036, holo; 118.8847). Rg on the other hand, is a parameter to describe the equilibrium conformation of a total system particularly in analyzing the proteins it is an indicative of the level of compaction in the structure, i.e. how the polypeptide chain is folded or unfolded [36]. Rg plot for Cα atoms and protein with time over the course of 10 ns simulations during apo and holo simulations is shown in the Fig. 8a-f and Fig. 9a-f. In the Rg plot for both Cα atoms and protein, we observed a notable fluctuation in MTs compared to the WT. Among the MTs, large deviations in Rg from the WT structure were observed during the apo and holo simulations of R110P (Table 4). These results indicate that compared to other MTs, p53C might have undergone a significant structural transition due to R110P.

**Figure 10:**
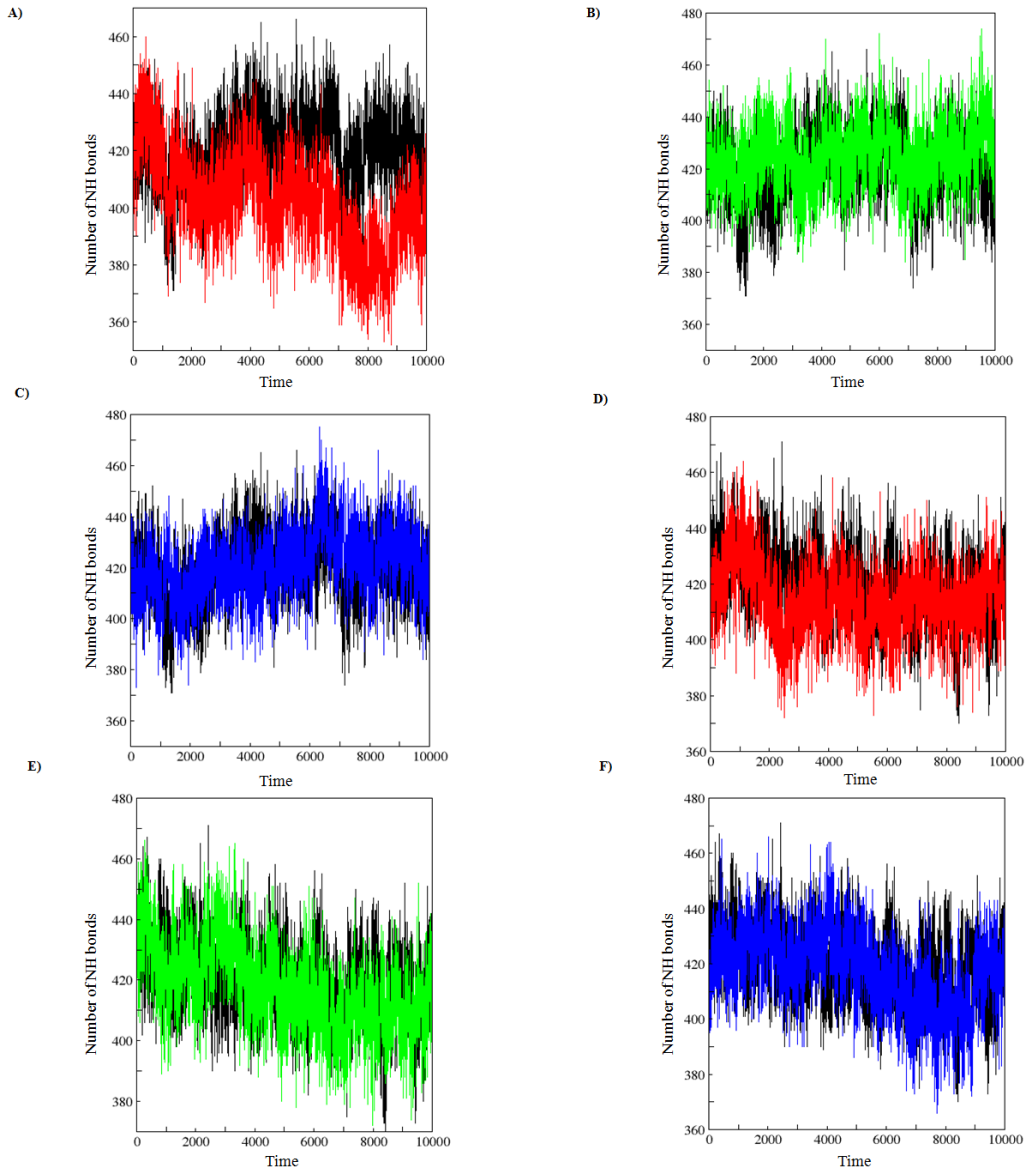
Average number of protein–solvent intermolecular hydrogen bonds in WT and MT p53C versus time. A), B), C) represent apo simulation D), E), F) represent holo simulation. Black: WT, red: R110P, green: P151T and blue: P278A.

**Figure 11:**
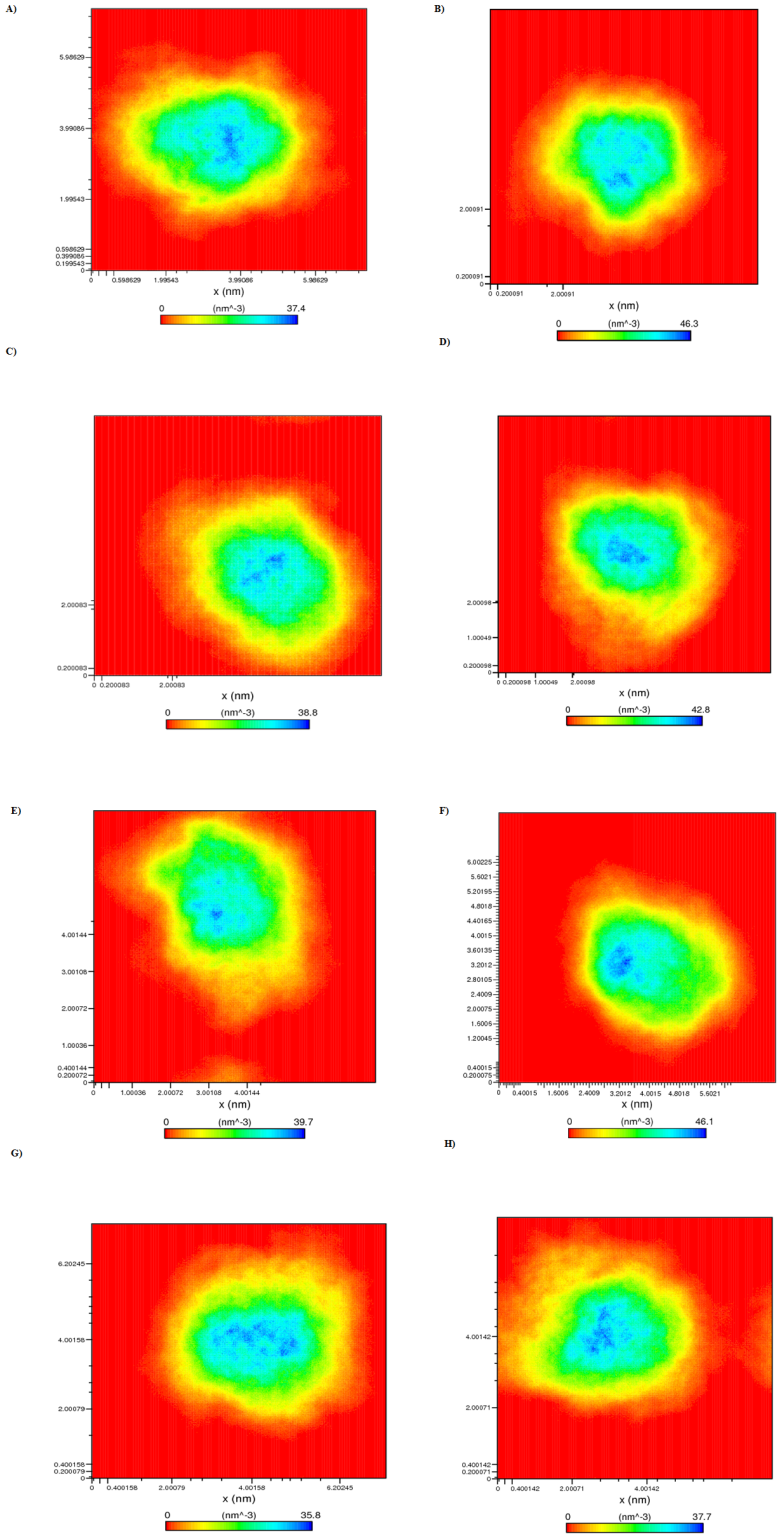
Number density plot of p53C. A) Apo WT B) Apo R110P C) Apo P151T D) Apo P278A E) Holo WT F) Holo R110P G) Holo P151T F) Holo P278A.

Further, one of the factors that accounts for maintaining the stable conformation of a protein is hydrogen bonding. In order to understand the reason for flexibility among the MTs we have performed the NH bond analysis of WT and MTs during apo and holo simulations and plotted in the Fig. 10a-f. Results showed a notable difference in protein-solvent intermolecular hydrogen bond pattern between the WT and MTs. Among the MTs, a decrease in the average number of hydrogen bonds was observed in R110P compared to the WT during both apo and holo simulations (Fig. 10a,d) indicting that the occurrence of this mutation may lead to a more flexible conformation in the presence or absence of Zn^2+^ at physiological conditions. However, the other MTs, P151T and P278A showed a decrease in average number of protein-solvent intermolecular hydrogen bonds only during holo simulations (Fig. 10e,f) indicating that these two mutants are flexible only in the presence of Zn^2+^ at physiological conditions. Further, we have plotted the atom density distribution to check if the MTs have caused any major changes in the orientation and atomic distribution. Results showed that atomic distribution of all the MTs were significantly differed from the WT in apo and holo simulations (Fig. 11a-h) indicating that all the MTs have a deleterious effect on the p53C.

**Figure 12:**
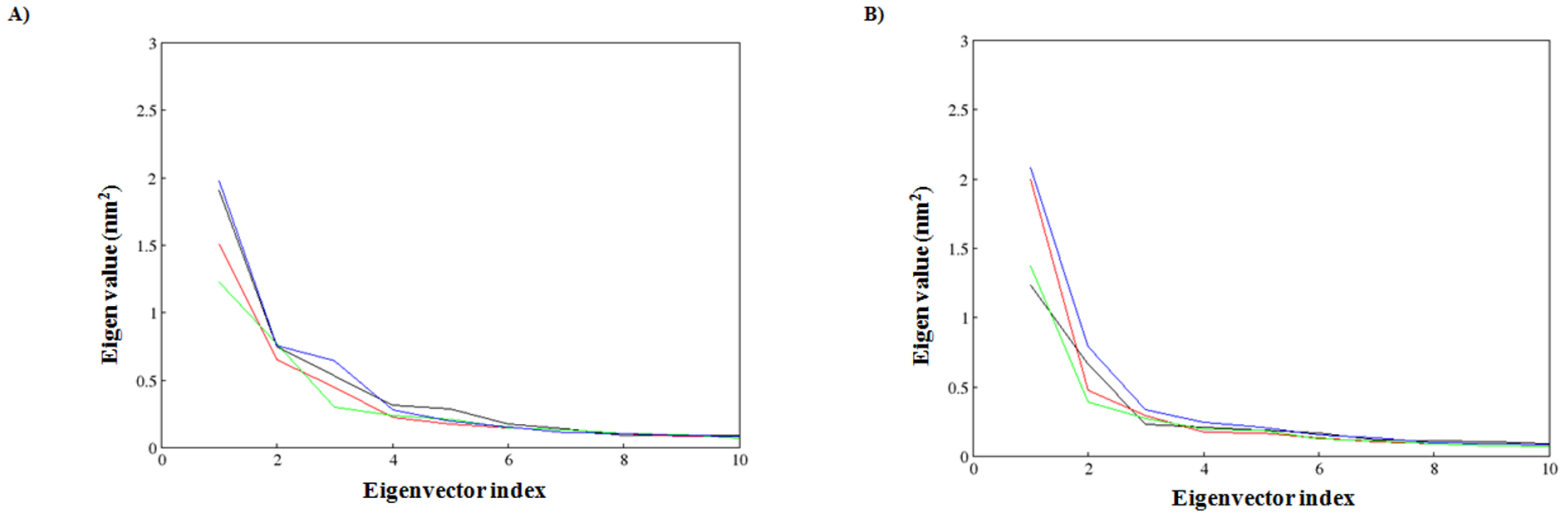
Plot of eigenvalues corresponding to eigenvector index for the first fifty modes of motion of p53C. A) represents the apo simulation B) represents the holo simulation. Black: WT, red: R110P, green: P151T and blue: P278A.

**Figure 13:**
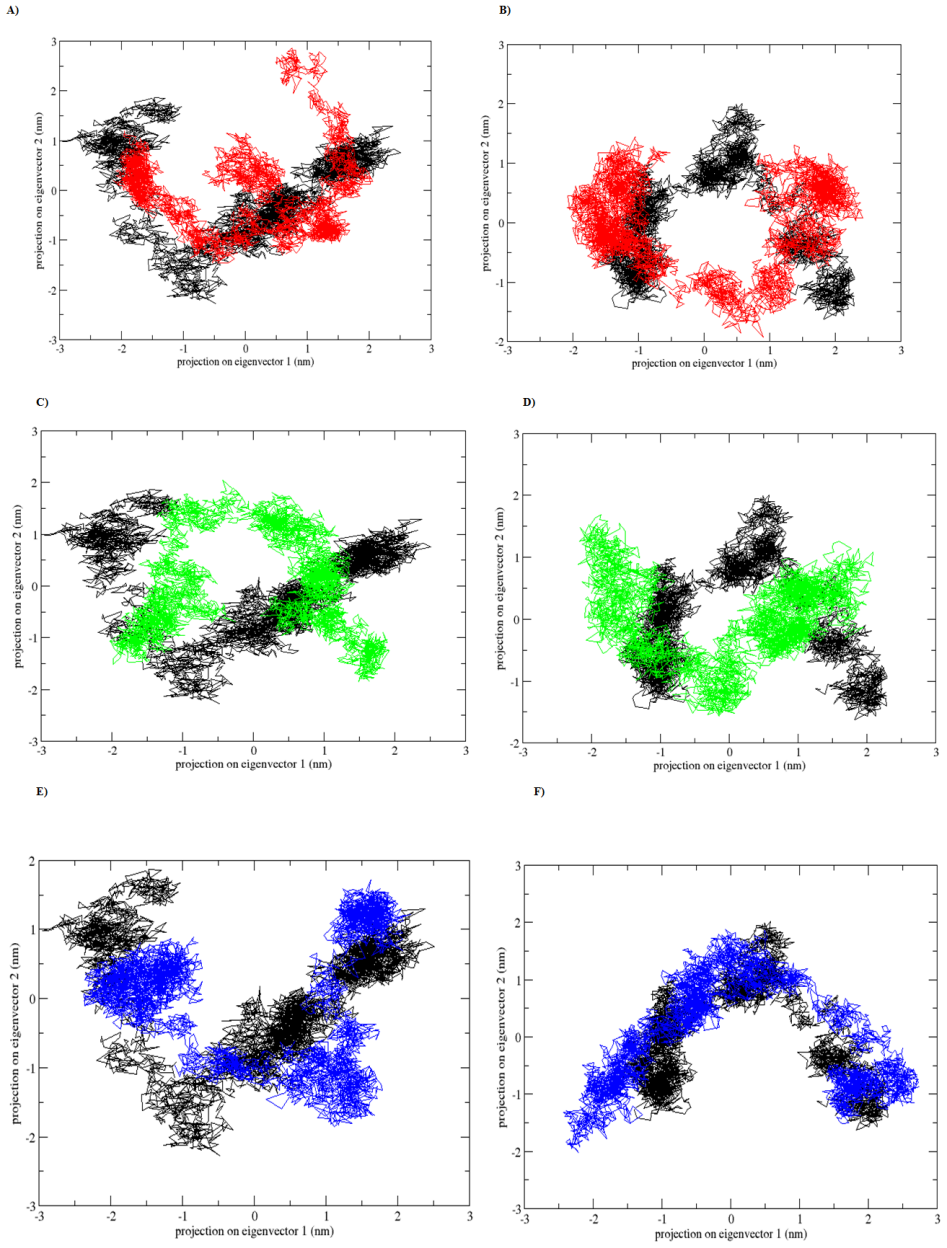
Projection of the motion of the p53C in the phase space along the first two principal eigenvectors. A), C), E) represents the apo simulation B), D), F) represents the holo simulation. Black: WT, red: R110P, green: P151T and blue: P278A.

Moreover, to identify the correlated motions of the WT and MTs during trajectory generated by apo and holo simulations and to support our MDS result we performed ED analysis. Since sum of the eigenvalues is a measure of the total motility in the system, we have plotted the eigenvalues against the corresponding eigenvector index for the first ten modes of motion at different trajectory lengths for WT and MTs during the apo and holo simulations in the Fig. 12 a, b. Only few eigenvectors showed large eigenvalues for both WT and MTs during the apo and holo simulations indicating that most of the internal motion of the protein is confined along small dimension in the essential subspace. The spectrum of eigenvalues in the Fig. 12 a,b indicated that major fluctuations of the system were confined to first two eigenvectors. Hence, the projection of trajectories of WT and MTs during the apo and holo simulations in the phase space along the first two principal components (PC1, PC2) at 300 K was plotted in the Fig. 13 a-f. Compared to apo simulation, during holo simulation MTs covered a larger region of phase space along PC1 and PC2 plane than WT. The overall flexibility of WT and MTs was calculated by the trace of the diagonalized covariance matrix of the Cα atomic positional fluctuations. Results from the trace of the covariance matrix (Table 4) confirmed the overall flexibility between MTs and WT at 300K during both apo and holo simulations. Overall the results reported from our study has confirmed that the substitution of Arginine at 110^th^ residue with Proline, Proline at 151^th^ residue with Threonine and Proline at 278^th^ residue with Alanine in the p53 core domain in the presence or absence of Zn^2+^ has an altered structure stability and essential hydrogen bond formation and thus these three mutants might play a significant role in initiating the susceptibility towards breast cancer. Further, our analysis indicates that compared to other MTs P151T and P278A, amino acid substitution of Arginine at 110^th^ residue with Proline (R110P) exhibit a highly deleterious effect on the p53C.

## Conclusion

The present study, will offer an in depth insight into the genotype–phenotype association of deleterious breast cancer associated nSNPs in *TP53*. Our study reports three mutations R110P, P151T and P278A associated with breast cancer phenotype and further the molecular dynamics revealed their respective major consequences on native p53 DNA-binding core domain. RMSD, Rg, SASA, NH bond and number density plots revealed their high deleterious nature on the p53 DNA-binding core domain in the presence and absence of Zn^2+^ ion. Overall, the present computational approach will provide a comprehensive view on destabilizing mechanisms of p53 SNPs in breast cancer and it may serve as a useful model for predicting the effect of SNPs in other proteins. The results reported in this study elucidate the role of deleterious mutations in p53 which may provide a useful information for the design of p53 mutants based therapeutic strategies against breast cancer.
